# Solubility of calcium silicate-based endodontic sealers in acidic and neutral pH using phosphate-free buffers

**DOI:** 10.1590/1807-3107bor-2026.vol40.078

**Published:** 2026-10-16

**Authors:** Cláudio Malizia Alves Ferreira, Juliana da Silva Chagas Cypriano, Brenda Xavier dos Santos, Vitória Cerqueira Soares, Karem Paula Pinto, Barbara de Paula Coelho, Luciana Moura Sassone, Emmanuel João Nogueira Leal da Silva

**Affiliations:** (a) Universidade do Estado do Rio de Janeiro – UERJ, School of Dentistry, Department of Integrated Clinical Procedures, Rio de Janeiro, RJ, Brazil.

**Keywords:** Hydrogen-Ion Concentration, Calcarea Silicata, Endodontics, Solubility, Root Canal Filling Materials

## Abstract

This study aimed to evaluate the solubility of calcium silicate-based endodontic sealers under acidic and neutral pH conditions using phosphate-free buffered solutions. The solubility of three calcium silicate-based sealers (BioRoot RCS, Bio-C Sealer, and AH Plus Bioceramic) and an epoxy resin-based sealer (AH Plus Jet) was evaluated according to ISO 6876:2012 guidelines, with modifications. Specimens (n = 12 per sealer) were immersed in phosphate-free buffered solutions at pH 5 and 7 and stored at 37°C for 24 hours, 7 days, and 30 days. Solubility was calculated as the percentage of initial mass loss. Statistical analyses were conducted using one-way ANOVA, Tukey's post hoc test, and independent t-tests (p < 0.05). AH Plus Jet met ISO 6876:2012 solubility standards at all time points, while all calcium silicate-based sealers exceeded the acceptable limit. Acidic pH significantly increased the solubility of these sealers (p < 0.05), except for BioRoot RCS at 30 days. Among the calcium silicate-based sealers, BioRoot RCS exhibited the lowest solubility, followed by Bio-C Sealer, whereas AH Plus Bioceramic showed the highest solubility and disintegrated after 7 days, preventing evaluation at 30 days. In contrast, AH Plus Jet remained stable under both pH conditions, exhibiting a slight mass gain over time. Overall, acidic pH increased the solubility of calcium silicate-based sealers, whereas AH Plus Jet remained stable and compliant with ISO 6876:2012 standards.

## Introduction

Calcium silicate-based endodontic sealers are widely recognized for their bioactivity, antimicrobial properties, and favorable setting ability in moist environments. However, concerns remain regarding their high solubility^1–3^ and potential setting failures,^3–5^ which may result from inadequate moisture balance,^6^ variations in pH,^7^ or interactions with intracanal medicaments.^8^ These factors can interfere with the hydration process and compromise mechanical properties. Such limitations could ultimately affect sealing ability and overall clinical performance.^9,10^

According to ANSI/ADA and ISO 6876/2012 standards, solubility is assessed by measuring mass loss after 24 hours of immersion in distilled water, with an acceptable limit of 3% and no visible disintegration. However, laboratory conditions differ significantly from clinical settings, where factors such as inflammation and bacterial byproducts can decrease pH.^11^ This acidic environment may negatively impact the performance of endodontic materials by increasing their solubility, compromising their sealing ability, and ultimately affecting their long-term stability.^12–14^ Given their direct interaction with living tissues and biological fluids, evaluating the impact of an acidic environment on root canal sealers is crucial.^15^ Despite this, research on how low pH influences the solubility of calcium silicate-based sealers remains limited.^7^

Although several studies have investigated sealer solubility under different immersion media and pH conditions, the available evidence is difficult to interpret because pH effects are often confounded by the chemical composition of the immersion solution. In particular, commonly used phosphate-containing media may introduce surface mineral precipitation that masks true material dissolution, whereas non-buffered solutions allow pH drift during testing, limiting the isolation of acidity as an independent variable.^7^ Consequently, the specific effect of pH on sealer solubility remains insufficiently delineated in the existing literature.

The release of calcium ions by calcium silicate-based sealers can lead to a rapid increase in the surrounding pH.^15^ Consequently, using buffered solutions in solubility assessments may help maintain a more stable pH environment in immersion fluids, allowing for a more accurate evaluation of pH influence on the physicochemical properties of these materials. A recent study demonstrated that an acidic environment reduced the short-term solubility of EndoSequence BC Sealer when immersed in phosphate-buffered saline (PBS), whereas its solubility remained unchanged in distilled water, a non-buffered solution.^7^ Additionally, immersion in phosphate-containing physiological solutions, such as PBS, promotes hydroxyapatite precipitation on the surface of calcium silicate-based sealers, potentially influencing their solubility over time.^7,16^ As a result, it remains challenging to isolate the direct role of acidity on solubility when phosphate-induced mineral deposition is present. Although such surface precipitation is often interpreted as bioactivity, it represents a physicochemical phenomenon that cannot be directly extrapolated to clinical conditions.^16^ Experimental models that stabilize pH while minimizing phosphate-related precipitation may therefore provide a clearer mechanistic assessment of how acidity influences sealer solubility, even if they do not reproduce the full biological complexity of the clinical environment.

Moreover, solubility should be interpreted as a surrogate physicochemical parameter rather than a direct predictor of long-term clinical performance. As recently emphasized by Silva and Versiani^17^ in an editorial, the growing reliance on isolated laboratory outcomes underscores the need for well-designed experimental models that deliberately isolate specific variables and clarify mechanistic behavior, rather than inferring clinical superiority from single parameters alone. In this context, controlled in vitro approaches remain essential to advance mechanistic understanding under defined conditions.

Given the limited evidence available, this study aimed to evaluate the feasibility of an in vitro experimental model using buffered immersion media – at acidic and neutral pH, and without phosphate ions – to assess the solubility of three calcium silicate-based root canal sealers: BioRoot RCS^®^ (Septodont, Saint-Maur-des-Fossés, France), Bio-C Sealer^®^ (Angelus, Londrina, Brazil), and AH Plus Bioceramic^®^ (Dentsply DeTrey, Konstanz, Germany), as well as an epoxy resin-based root canal sealer (AH Plus Jet^®^, Dentsply DeTrey, Konstanz, Germany). The null hypotheses tested were: a) no significant differences in solubility would be observed among the sealers, and b) acidic pH would not influence solubility.

## Methods

The manuscript for this laboratory study was prepared according to the Preferred Reporting Items for Laboratory studies in Endodontology (PRILE) 2021 guidelines (Figure 1).

**Figure 1:**
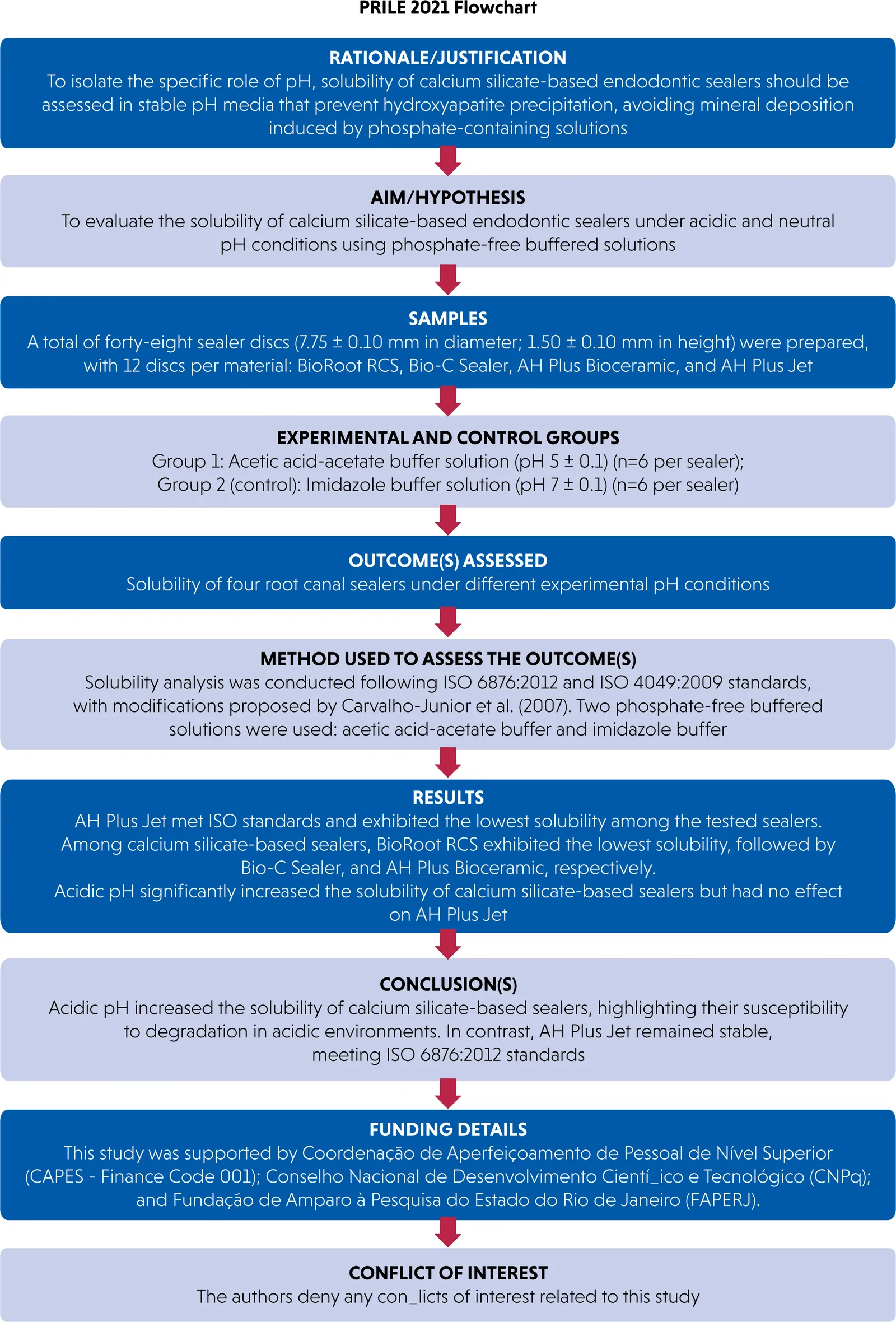
PRILE Flowchart.

### Sample size calculation

A power analysis was performed using G*Power 3.1 software (Heinrich Heine University, Dusseldorf, Germany) based on data from previous studies.^3,18^ The fixed effects ANOVA, omnibus, one-way test was selected from the F tests family for an analysis with α = 5%, 95% power, and an effect size of 1.03. A total of forty-eight specimens (n = 6 per group) was determined as the ideal sample size to detect significant differences.

### Specimen preparation

The root canal sealers were prepared according to the manufacturers’ instructions. The manufacturers, lot numbers, compositions, and mixing proportions for each material are detailed in Table 1.

**Table 1:** Root canal sealers and their lot numbers, manufacturers, compositions and proportions.

Sealer/Lot number	Manufacturer	Composition	Proportion
AH Plus Bioceramic (KI220616)	Dentsply De Trey Gmbh, Konstanz, Germany	Tricalcium silicate, zirconium dioxide, dimethyl sulfoxide, lithium carbonate, bentonitic clay, polyvinyl alcohol, polyvinylpyrrolidone.	Ready to use
BioRoot RCS (B25218)	Septodont, Saint-Maur-des-Fossés Cedex, France	Powder (tricalcium silicate, zirconium oxide, povidone); Liquid (dihydrated calcium chloride, areo, purified water).	Powder: measuring spoon; Liquid: 1 single-dose ampoule of 0.20 mL.
Bio-C Sealer (59660)	Brasseler USA, Savannah, USA	Zirconium oxide, calcium silicates, monobasic calcium phosphate, calcium hydroxide, filling and thickening agents.	Ready to use
AH Plus Jet (1806000082)	Dentsply De Trey Gmbh, Konstanz, Germany	Bisphenol A/F epoxy resin, calcium tungstate, zirconium oxide, silica, iron oxide pigments, dibenzylamine, aminoadamantane, silicone oil.	1g:1g (Paste/Paste) automix

Solubility analyses were conducted following ISO 6876:2012 standard,^19^ with modifications.^20^ A total of 48 silicone molds (7.75 ± 0.10 mm in diameter; 1.50 ± 0.10 mm in height) were prepared for specimen fabrication (n = 12 per sealer). Before filling, a nylon thread (0.2 mm thick, 8 cm long) was placed in each mold.

AH Plus Jet was dispensed into the molds using a mixing tip attached to the syringe. BioRoot RCS, after mixing, was placed into the molds with a resin spatula. Bio-C Sealer and AH Plus Bioceramic were directly dispensed into the molds using plastic applicator tips from their respective syringes (Figure 2). To facilitate the setting reaction, 2 μL of distilled water was added to each mold, following ISO 6876:2012 guidelines.

**Figure 2:**
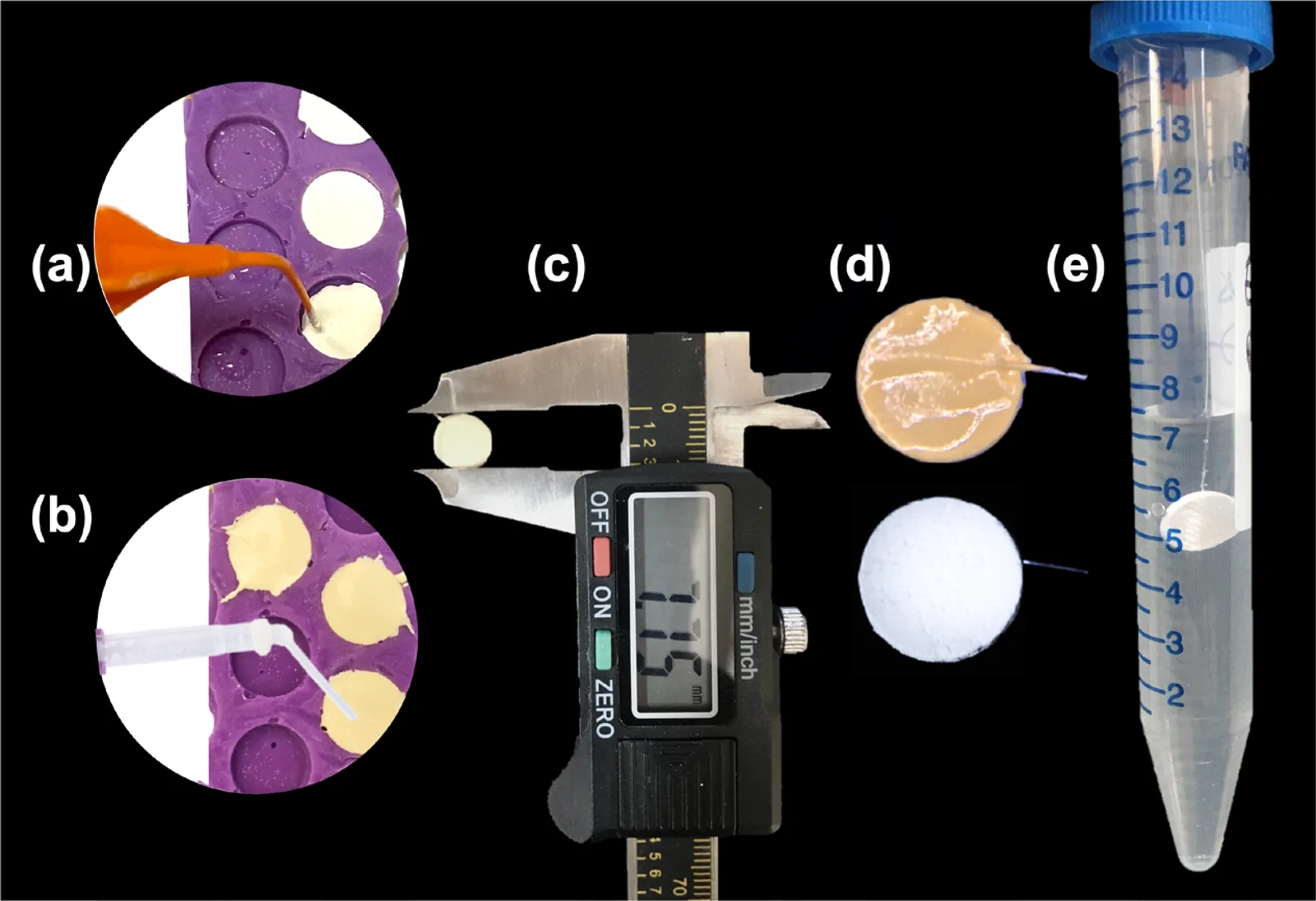
Schematic representation of specimen preparation and testing procedures.

(a, b) Placement of root canal sealers into silicone molds. (c) Measurement of specimen diameter. (d) Sealer specimens after setting. (e) Sealer specimen immersed in the phosphate-free buffered immersion media for solubility assessment.

After filling the molds, the specimens were incubated at 37°C with 95% relative humidity for 50% longer than the manufacturer-specified setting time (approximately 24 hours for AH Plus Jet and 4 hours for BioRoot RCS, Bio-C Sealer, and AH Plus Bioceramic). To standardize the setting conditions, all specimens were incubated for 48 hours. After incubation, the specimens were removed, and surface irregularities were smoothed using a scalpel blade. Each specimen was then weighed three times on an electronic analytical balance to determine the average initial mass (IM).

### Solubility test

The specimens were then suspended by the nylon thread, ensuring no contact with the container walls, and individually immersed in hermetically sealed Falcon tubes containing 7.5 mL of immersion media (Figure 2). Subsequently, they were stored at 37 °C for 24 hours, 7 days, and 30 days. The immersion medium conditions included:

- Acetic acid-acetate buffer (acetic acid / sodium acetate) – 0.1 mol L^−1^, pH 5 (n = 6 per sealer); and
- Imidazole buffer (imidazole / hydrochloric acid) – 0.05 mol L^−1^, pH 7 (n = 6 per sealer).

The pH was measured using a calibrated pH meter with standard buffer solutions at pH 4, 7, and 10. Adjustments were made as needed using 0.5 mol L^−1^ NaOH (Sigma-Aldrich, St. Louis, USA) or 0.1 mol L^−1^ HCl (Sigma-Aldrich, St. Louis, USA) at a controlled room temperature of 25°C.

After each immersion period, the specimens were removed, rinsed with 2 mL of distilled water, and placed in a desiccator containing silica gel for 24 hours. They were then weighed three times to determine the average final mass (FM). Solubility was calculated as a percentage of the initial mass using the formula:


$$S=\frac{IM-FM}{IM}x100\%$$

This process was repeated for each specimen at the end of each immersion period, with cumulative evaluations conducted at 7 and 30 days. After each measurement, the specimens were re-immersed in the Falcon tubes and stored at 37°C (Figure 3). The immersion media were not replaced during the experiment; instead, the pH of all flasks was monitored and adjusted every 48 hours throughout the 30-day period. During this time, pH variation in the immersion media remained within ± 0.2 units, confirming stable pH conditions under both acidic and neutral environments. According to ISO 6876:2012, the solubility of endodontic materials must not exceed 3% of their mass and should exhibit no visible signs of disintegration.

**Figure 3:**
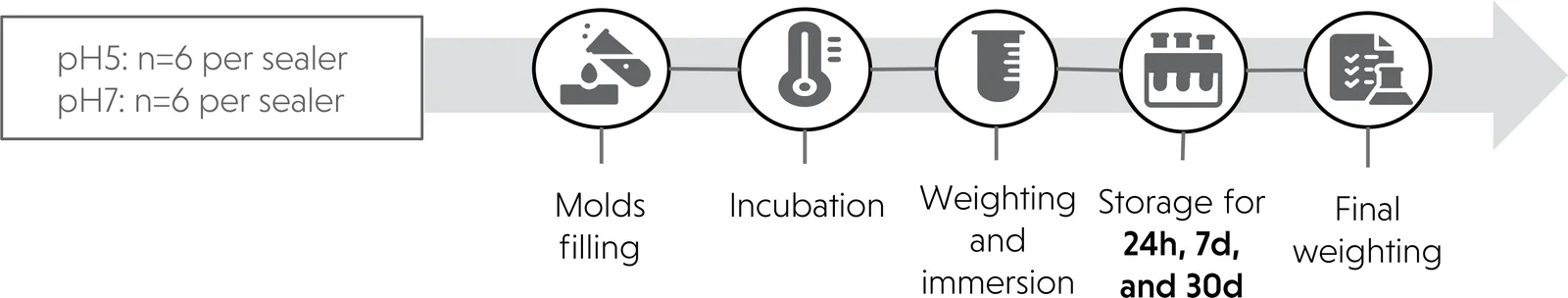
Flowchart of the experimental stages.

### Statistical analysis

The normal distribution of solubility data was confirmed by the Shapiro-Wilk test (p > 0.05). Statistical analyses were conducted at a 5% significance level using Jamovi^®^ software (Version 2.3.18.0, Sydney, Australia). One-way ANOVA followed by Tukey's post hoc test was used to compare the solubility of different sealers under the same experimental time and pH conditions. A repeated measures ANOVA with Bonferroni's post hoc adjustment was applied to assess the effect of time on solubility, accounting for within-specimen correlation across time-points. These analyses were performed separately for each pH condition. An independent t-test was performed to evaluate the influence of different pH conditions on the same sealer at a given experimental time.

## Results

The results indicated that AH Plus Jet met ISO standards and exhibited the lowest solubility among the tested sealers (p < 0.05). Among the calcium silicate-based sealers, BioRoot RCS showed the lowest solubility (p < 0.05). Bio-C Sealer had higher solubility than BioRoot RCS (p < 0.05), except at 30 days in pH 7 (p > 0.05), where both had comparable solubility. AH Plus Bioceramic displayed the highest solubility (p < 0.05), especially after 24 hours under both pH conditions and after 7 days under acidic pH. However, its solubility at 30 days could not be assessed due to sample disintegration (Table 2 and Figure 4).

**Table 2:** Solubility (% mass loss) of root canal sealers after storage in phosphate-free buffered immersion media (mean ± standard deviation).

Variables		24h	7d	30d
Bio-C Sealer				
	pH 5	21.25± 1.25^A1^	31.28± 1.42^B1^	43.73± 1.93^C1^
	pH 7	**17.52± 0.94** ^**A1**^	**28.76± 1.10** ^**B1**^	**38.80± 1.03** ^**C1**^
BioRoot RCS				
	pH 5	15.78 ± 1.98^A2^	24.35 ± 1.79^B2^	37.89 ± 3.85^C2^
	pH 7	**11.68 ± 1.40** ^**A2**^	**16.77 ± 2.22** ^**A2**^	34.41± 4.49^B1^
AH Plus Jet				
	pH 5	0.05 ± 0.12^A3^	-0.13 ± 0.19^AB3^	-0.17 ± 0.14^B3^
	pH 7	0.08 ± 0.11^A3^	-0.04 ± 0.19^A3^	-0.14 ± 0.07^A2^
AH Plus Bioceramic				
	pH 5	26.02 ± 2.68^A4^	36.52 ± 6.25^B4^	*
	pH 7	**20.90 ± 2.94** ^**A4**^	**28.67 ± 5.35** ^**B4**^	*

Different superscript letters indicate significant differences for the same sealer, pH condition, and different evaluation periods (p < 0.05). Bold text indicates significant differences for the same sealer, at the same experimental time, and different pH conditions (p < 0.05). Different superscript numbers indicate significant differences for different sealers under the same pH conditions and the same experimental time (p < 0.05).

* Solubility could not be evaluated due to sample disintegration.

**Figure 4:**
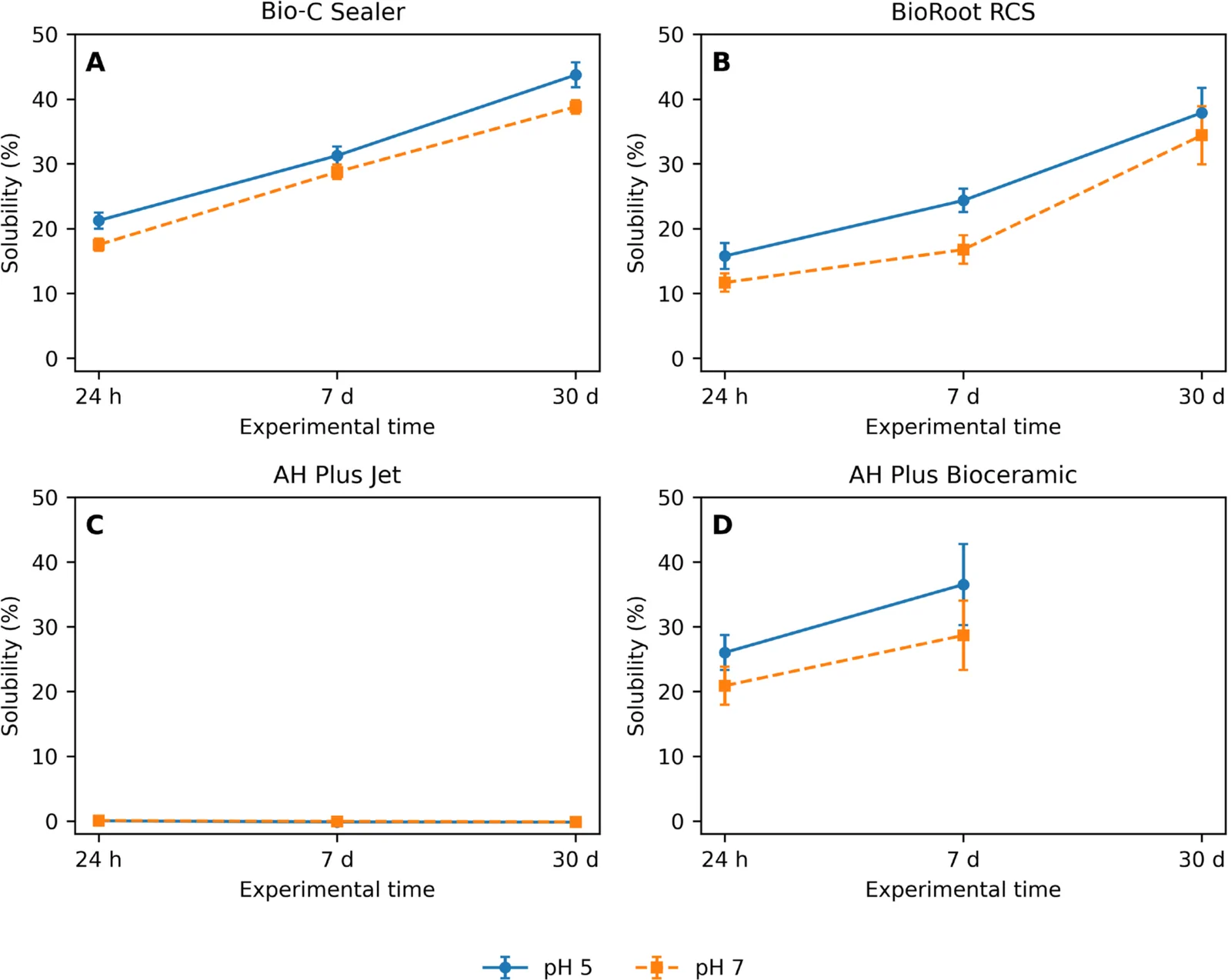
Solubility (%) of root canal sealers over time under acidic (pH 5) and neutral (pH 7) conditions.

(A) Bio-C Sealer, (B) BioRoot RCS, (C) AH Plus Jet, and (D) AH Plus Bioceramic. Data are presented as mean ± standard deviation. The absence of data at 30 days for AH Plus Bioceramic reflects specimen disintegration.

Over time, all calcium silicate-based sealers showed increased solubility, with none meeting ISO standards at any time point or under any pH condition (p < 0.05). In contrast, AH Plus Jet showed no significant change in solubility at pH 7 (p > 0.05), while a significant mass gain was observed over time under acidic conditions (pH 5) (p < 0.05).

Acidic conditions generally increased the solubility of calcium silicate-based sealers, except for BioRoot RCS at 30 days (p < 0.05). Meanwhile, AH Plus Jet was unaffected by pH variations.

## Discussion

This study investigated the effect of acidic pH on the solubility of three calcium silicate-based root canal sealers (BioRoot RCS, Bio-C Sealer, and AH Plus Bioceramic) compared with AH Plus Jet. The results showed that AH Plus Jet consistently met the ISO 6876:2012 requirements under all pH conditions and time intervals, whereas none of the calcium silicate-based sealers met the ISO criteria at any pH or time point. Among the materials tested, AH Plus Jet had the lowest solubility, followed by BioRoot RCS and Bio-C Sealer, with AH Plus Bioceramic showing the highest solubility, leading to rejection of the first null hypothesis. These findings closely align with previous studies reporting significantly higher solubility for calcium silicate-based sealers compared with epoxy resin–based materials.^1,3,16,18,21–24^

Rather than merely confirming the greater physicochemical stability of epoxy resin-based sealers, the present findings provide a more controlled interpretation of how acidic conditions selectively compromise calcium silicate-based materials when pH is isolated from confounding surface precipitation phenomena. Although the bioactivity and calcium ion release of calcium silicate-based sealers are often considered advantageous, the present findings reinforce earlier reports indicating that their compliance with ISO 6876:2012 solubility limits remains inconsistent and limited.^25–28^ The progressive increase in solubility observed for all calcium silicate-based sealers over time further corroborates previous long-term investigations that highlighted their susceptibility to material degradation in aqueous environments.^16,21,22^ In contrast, the mass gain observed for AH Plus Jet aligns with earlier reports attributing this behavior to water sorption within the epoxy resin matrix, which may partially offset material loss and result in slight volumetric expansion;^29^ however, an alternative explanation is the precipitation of buffer salts onto the specimen surface during the desiccation process, even though all specimens were rinsed with 2 mL of distilled water prior to drying. This possibility cannot be fully excluded in the absence of specific controls to quantify surface deposition. This distinction is important from a translational standpoint, as it underscores that apparent mass stability does not necessarily reflect true chemical inertness, but may also result from surface phenomena dependent on the immersion protocol.

The superior stability of AH Plus Jet observed in the present study is consistent with its well-documented stable polymeric structure, in which bisphenol epoxy resin, and extensive cross-linking formed during polymerization, confer chemical resistance and limit degradation.^18,30^ On the other hand, the elevated solubility of calcium silicate-based sealers may be explained by mechanisms previously described in the literature, including the presence of nanosized hydrophilic particles in their composition, the dissociation of calcium hydroxide formed during material hydration, and the existence of weaker internal chemical bonds that facilitate material dissolution.^15,16,21,31,32^ Notably, the magnitude of solubility values observed in the present study is comparable to those previously reported, supporting the consistency of these findings with existing evidence.^1,3,7,16,18,21–24^ Importantly, the present model allows these mechanisms to be interpreted under stabilized pH conditions, thereby strengthening the mechanistic link between acidity and material dissolution rather than attributing solubility changes to secondary mineral deposition.

Importantly, this does not invalidate the model but rather highlights its ability to discriminate differential susceptibility to acidic challenge under controlled conditions. Although the phosphate-free buffered model was designed to isolate the effect of acidity and minimize surface precipitation artifacts, it cannot fully dissociate material-inherent dissolution from environment-driven acceleration of degradation. Consequently, the magnitude of mass loss should be interpreted in relative rather than absolute terms, reflecting differential susceptibility to acidic conditions rather than direct clinical equivalence.

The calcium silicate-based sealers evaluated in this study were selected for their distinct formulations and hydration mechanisms. Bio-C Sealer and AH Plus Bioceramic are premixed, ready-to-use materials that depend on environmental moisture for hydration, whereas BioRoot RCS is supplied as a powder-liquid formulation, allowing more controlled hydration conditions.^6,21^ This distinction may explain the comparatively lower solubility of BioRoot RCS observed in this study, supporting previous findings that powder-liquid formulations exhibit greater physicochemical stability due to more predictable moisture availability during setting.^6^ Although all calcium silicate-based sealers evaluated contain zirconium oxide as a radiopacifier—a component previously associated with improved stability and reduced solubility^25,33^ —this effect did not uniformly translate into improved performance. Notably, AH Plus Bioceramic, despite containing a high proportion of zirconium dioxide (50–70%), exhibited the greatest solubility and disintegrated after seven days, precluding its evaluation at the 30-day time point. This behavior is consistent with previous reports^22^ and may be attributed to its relatively low tricalcium silicate content (5–15%), a phase considered critical for maintaining structural integrity and long-term physicochemical stability.^22,24^

Regarding the influence of pH, the present results showed that acidic conditions significantly increased the solubility of all calcium silicate-based sealers except for BioRoot RCS at 30 days, whereas AH Plus Jet was unaffected by pH variations. These findings led to the rejection of the second null hypothesis and partially contrast with previous studies reporting no significant differences between acidic and neutral conditions for epoxy resin and calcium silicate-based sealers. This apparent discrepancy highlights the translational relevance of the immersion model used, as methodological differences – particularly the chemical composition of the immersion media – can substantially influence the interpretation of solubility outcomes. While distilled water lacks buffering capacity and allows substantial pH fluctuations, PBS promotes hydroxyapatite precipitation on calcium silicate-based sealer surfaces, which may artificially reduce apparent solubility by increasing specimen mass.^7,15 16 31^

To better simulate the periapical environment, alternative immersion media to distilled water have been proposed;^18,34^ however, phosphate-containing solutions such as PBS may introduce additional confounding factors, as phosphate ions can induce hydroxyapatite precipitation on calcium silicate-based sealers.^7,16,31^ Moreover, the hydration reactions and bioactivity observed for these materials under in vitro conditions may differ from those occurring in the presence of dentinal fluid, so their bioactivity cannot be assumed to directly translate to the clinical setting.^35^ By employing phosphate-free buffered immersion media, the present study isolates acidity as an experimental variable, providing mechanistic insight into how acidic environments – such as those associated with periapical inflammation and bacterial metabolism – may compromise material stability independently of surface precipitation phenomena.

Nevertheless, the limitations of this *in vitro* model must be acknowledged. Although ISO methodology recommends solubility testing by immersing sealer specimens in distilled water, this laboratory setup does not fully replicate the complexity of the clinical environment, where dynamic fluid exchange, enzymatic activity, inflammation, and bacterial byproducts can alter local pH and influence the physicochemical behavior of sealers.^15,28,36^ Furthermore, assessing solubility solely on the basis of mass loss, without complementary surface or volumetric analyses, restricts interpretation of the underlying degradation mechanisms. Accordingly, solubility should be viewed as a mechanistic indicator rather than a direct surrogate for long-term clinical performance, in line with recent editorial discussions emphasizing the limitations of isolated laboratory endpoints.^17^

Future investigations should therefore focus on developing experimental models that better simulate the endodontic environment, incorporating biological and mechanical variables such as salivary components, inflammatory mediators, and cyclic mechanical stress to improve clinical relevance. Translational studies evaluating the long-term performance of calcium silicate-based sealers *in vivo* are also warranted to bridge the gap between laboratory findings and real-world applications. From a translational perspective, the pronounced solubility of calcium silicate-based sealers under acidic conditions observed in this study suggests that material stability may be compromised in inflamed or acidic periapical environments, reinforcing the need for careful material selection and cautious interpretation of *in vitro* bioactivity claims.

## Conclusion

AH Plus Jet met the solubility standards established by ISO 6876:2012, whereas Bio-C Sealer and BioRoot RCS showed significantly higher solubility at all time points and did not meet the required criteria. Among the tested materials, AH Plus Bioceramic had the highest solubility and completely disintegrated after 7 days. Exposure to acidic pH significantly increased the solubility of calcium silicate-based sealers, highlighting their susceptibility to acidic environments.

## Data Availability

The datasets generated during and/or analyzed during the current study are available from the corresponding author on reasonable request.
